# Pharmacokinetic Developability and Disposition Profiles of Bispecific Antibodies: A Case Study with Two Molecules

**DOI:** 10.3390/antib11010002

**Published:** 2021-12-28

**Authors:** Amita Datta-Mannan, Robin Brown, Stephanie Key, Paul Cain, Yiqing Feng

**Affiliations:** 1 Department of Exploratory Medicine and Pharmacology, Lilly Research Laboratories, Lilly Corporate Center, Indianapolis, IN 46225, USA; 2 Department of Drug Disposition, Lilly Research Laboratories, Lilly Corporate Center, Indianapolis, IN 46225, USA; brown_robin_m@lilly.com (R.B.); key_stephanie_l@lilly.com (S.K.); 3 Lilly Research Laboratories, Lilly Technology Center North, Biotechnology Discovery Research, Indianapolis, IN 46221, USA; cain_paul_f@lilly.com (P.C.); feng_yiqing@lilly.com (Y.F.)

**Keywords:** bispecific antibody, monoclonal antibody, scFv, single-chain variable fragment, pharmacokinetic, FcRn neonatal Fc receptor, physiochemical properties

## Abstract

Bispecific antibodies (BsAb) that engage multiple pathways are a promising therapeutic strategy to improve and prolong the efficacy of biologics in complex diseases. In the early stages of discovery, BsAbs often exhibit a broad range of pharmacokinetic (PK) behavior. Optimization of the neonatal Fc receptor (FcRn) interactions and removal of undesirable physiochemical properties have been used to improve the ‘pharmacokinetic developability’ for various monoclonal antibody (mAb) therapeutics, yet there is a sparsity of such information for BsAbs. The present work evaluated the influence of FcRn interactions and inherent physiochemical properties on the PK of two related single chain variable fragment (scFv)-based BsAbs. Despite their close relation, the two BsAbs exhibit disparate PK in cynomolgus monkeys with BsAb-1 having an aberrant clearance of ~2 mL/h/kg and BsAb-2 displaying a an ~10-fold slower clearance (~0.2 mL/h/kg). Evaluation of the physiochemical characteristics of the molecules, including charge, non-specific binding, thermal stability, and hydrophobic properties, as well as FcRn interactions showed some differences. In-depth drug disposition results revealed that a substantial disparity in the complete release from FcRn at a neutral pH is a primary factor contributing to the rapid clearance of the BsAb-1 while other biophysical characteristics were largely comparable between molecules.

## 1. Introduction

With three approvals and another ~100 in clinical development, bispecific antibodies (BsAbs) represent an important class of therapeutic modalities [1,2]. The intent of BsAb therapy is for a single molecule to interfere with multiple disease pathways by recognizing two different epitopes or antigens. These interactions can expand and prolong the efficacy of these modalities in complex disease indications. Another attractive quality of BsAbs is their potential to provide novel functionalities that do not exist in mixtures of the parental antibodies leading to synergistic biological effects. Given that many disorders including cancer, metabolic diseases (including diabetes and cardiovascular illnesses), and autoimmune diseases display multiple and/or redundant mechanisms that fuel their progression, BsAbs have the potential to provide increasingly effective therapeutic options to patients compared with antibodies and other therapeutic entities that interact or modulate the activity of a single target [3,4,5]. Innovation in the field of protein engineering and advancements in technology have led to the design of over 100 BsAb formats [6]. While some BsAbs are simply smaller proteins comprised of two linked antigen-binding fragments, a number of other BsAbs formats leverage the basic modular nature of the IgG structure. The IgG-like BsAb molecules consist of subunits on individual antibodies attached to an agonistic/antagonistic mAb that impart the ability to bind dual soluble or membrane bound ligands or a combination of both. These formats include DVD-Ig, cross-mAbs, IgG-extracellular domain (ECD), and IgG-scFv constructs [6,7].

Despite their exceptional therapeutic promise and structural tractability, the translation of BsAbs as medicines has been relatively slow compared with mAbs [2]. For example, the dual activity of T cell redirection and engagement was described approximately >30 years prior to the 2009 launch of catumaxomab (withdrawn in 2017 for commercial reasons) and more recently blinatumomab (approved 2017) and amivantamab (approved 2021) both for the treatment of cancer. The first BsAb approved outside of oncology is emicizumab for the treatment of hemophilia which also occurred more recently in 2017. Similar to most antibody therapeutics, the causalities of the slow clinical success for BsAbs can be generally related to several factors, including an incomplete understanding of the biological mechanism of action, poorly defined exposure-response profiles, insufficient safety margins, strategic industry decisions and immunogenicity. The increased inherent structural diversity and tractability BsAbs afford relative to mAbs also leads to greater potential for uncertainty in their pharmacokinetic and disposition profiles. Thus, in addition to the aforementioned challenges, unpredicted aberrant pharmacokinetic profiles requiring increased empirical protein engineering can also limit the potential advantages BsAbs offer pharmacologically relative to classical monospecific mAbs. 

**Figure 1:**
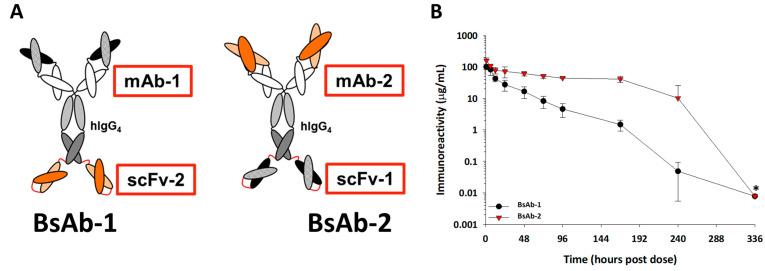
(**A**) Cartoon representations of the BsAb-1 and BsAb-2. BsAb-1 and BsAb-2 were constructed with scFv-2 and scFv-1, respectfully, covalently linked to the C-terminal end of the HC of the mAb-1 and mAb-2, respectfully, using Glycine-Serine linkers (shown in red). The BsAb molecules consist of a target binding orientation switch. The Fab region of BsAb-1 (consisting of mAb-1) binds to the same ligand as the scFv (scFv-1) component of BsAb-2; the Fab region of BsAb-2 (consisting of mAb-2) binds the same ligand as the scFv-2 region of BsAb-1. In the cartoon representation, the dark and light orange structures represent the antigen binding regions of the scFv-2 and Fab portions of mAb-2, whereas the dark and light black structures represent antigen binding regions of the scFv-1 and Fab portions of mAb-1. (**B**) The mean pharmacokinetic profiles of BsAb-1 and BsAb-2 following a single 5 mg/ kg IV administration to cynomolgus monkeys. Data are the mean for three animals/timepoint for each molecule. * Excluded from PK parameter calculations due to positive anti-drug antibody titers.

As a means to mitigate poor pharmacokinetics for mAbs, several studies have reported leveraging preclinical in vivo and in vitro physiochemical characterization-based PK developability strategies during the discovery process [8,9,10]. These approaches have been used to improve the probability of success by selecting or engineering mAbs with increased stability (physical, chemical, and thermal stabilities) and lower non-specific or unintended interactions [11,12]. Improving the stability and lowering the risk of unintended interactions, in turn provides enhanced human exposure profiles to support the intended dose and frequency of administration. Indeed, we and other groups reported connecting preclinical pharmacokinetics with various physiochemical characterization along with an FcRn interaction analyses in an integrated manner to inform the selection and engineering of mAbs with optimized pharmacokinetic profiles [11,12,13,14,15,16,17]. Our laboratories extended these approaches to some BsAbs that utilize IgG-ECD and IgG-scFv formats [11,14]. These studies revealed that poor physiochemical properties in some BsAb formats contributed to an increased clearance rate, driven by endothelial cell-based association/clearance mechanisms in the liver; moreover, the studies showed that engineering the structural configuration of the ECD mitigated aberrant pharmacokinetic behavior of the BsAbs. While these initial studies lay an important foundation for understanding non-target-related factors influencing the disposition and pharmacokinetics of BsAbs, there remains a paucity of data and an incomplete understanding of the balance between the in vitro physiochemical factors and in vivo physiological mechanisms that influence the peripheral clearance and disposition of BsAbs. Moreover, while previous studies were able to connect physiochemical properties to IgG-ECD BsAb pharmacokinetics in a post-hoc analysis, there remains considerable opportunity to define the relative contribution of the various non-target-related factors influencing the non-specific clearance of another BsAb format in an a priori manner. With these points in mind, we designed the present study to evaluate the physiochemical properties and connectivity of these with in vivo mechanism(s) involved in the clearance of two IgG-scFv constructs (deemed BsAb-1 and BsAb-2; Figure 1A) using preclinical models. 

The IgG-scFv constructs were fabricated with scFv units and mAbs targeting two distinct soluble ligands having minimal peripheral concentrations in normal animals, so that the in vivo kinetics and disposition can be evaluated in the absence of target mediated drug disposition (TMDD). The Fab (Fab-1) region of BsAb-1 binds to the same ligand as the scFv (scFv-1) component of BsAb-2 and relatedly, the Fab-2 region of BsAb-2 binds to the same target as scFv-2 in BsAb-1 (Figure 1A). While both BsAbs orientations were imparted with the same antigen binding properties, we observed rapid clearance (~2 mL/h/kg) of BsAb-1 and acceptable clearance (~0.2 mL/h/kg) of BsAb-2 in cynomolgus monkeys. The characterization of the two BsAbs revealed small differences in physical and thermal stability profiles yet a substantial difference in the FcRn-based release at neutral pH. The evaluation of the biodistribution of the two molecules in cynomolgus monkeys indicated distribution to the same organs to the same quantitative extent, but BsAb-1 was more rapidly cleared from tissue. Taken together, the in vitro and in vivo data indicate that the poor release of BsAb-1 from FcRn at a neutral pH is a major contributor to its aberrant clearance in cynomolgus monkeys. The observation is mechanistically distinct from the proposed increased hydrophobic interaction findings alone that led to aberrant kinetics observed for other BsAb formats in the earlier studies, highlighting the complexity of the issue [11,14]. The findings in this report confirm the need for continued evaluation and delineation of the balance between factors influencing the disposition and pharmacokinetics of various BsAbs and the interplay of the BsAb format on these parameters.

## 2. Materials and Methods

### 2.1. Expression and Purification of Recombinant BsAbs and Cynomolgus Monkey FcRn

The BsAbs used in this study were expressed in either transient HEK-293 or stably transfected Chinese hamster ovary (CHO) cells and purified to be >95% purity. Recombinant, soluble cynomolgus monkey FcRn was generated as previously described [18,19].

### 2.2. Solubility Determination

To assess their relative solubility, the BsAbs were concentrated to 20 mg/mL in the PBS buffer using 100 K MWCO ultrafiltration concentrators. Samples were stored at 5 °C for 10 days prior to visual inspection and turbidity assessment. Turbidity was determined by absorbance at 550 nm calibrated to a standard curve of turbidity NTU calibration standards (Sigma-Aldrich, St. Louis, MO, USA). Following the 10-day at 5 °C hold, samples were stored for an additional 7 days at 37 °C and assessed visually and for turbidity by absorbance at 550 nm.

### 2.3. Isoelectric Points Determination

The isoelectric points were measured or calculated as described previously [20]. The pI of each the Fab and scFv was calculated from the primary sequences using algorithm in Molecular Operating Environment (MOE2019) (Chemical Computing Group, Montreal, Canada) and outlined by Sillero and colleagues [21].

### 2.4. Hydrophobic Interaction Chromatography

The overall hydrophobicity of the BsAbs was assessed by hydrophobic interaction chromatography (HIC-HPLC). Samples were diluted to 1 mg/mL in PBS and injected onto a TSKgel Butyl-NPR HIC column (Tosoh Cat.# 0014947; Tosho Biosciences, King of Prussia, PA, USA) preequilibrated in 25 mM potassium phosphate, 1.5 M ammonium sulfate, and pH 6.8. Samples were eluted with a 20-min linear gradient into 25 mM potassium phosphate, pH 6.8, 20% isopropanol. 

### 2.5. Differential Scanning Calorimetry Analysis

Thermal stability of the BsAbs was assessed by DSC. Samples were prepared at 1 mg/mL in PBS. The thermal transition of each antibody was measured using an automated MicroCal PEAQ-DSC (Malvern Panalytical, Malvern, UK) in a scan rate of 1 °C/min for the temperature range of 20–110 °C, after equilibration at 20 °C for 3 min. Thermograms for each sample were buffer-referenced and baseline-subtracted. 

### 2.6. Heparin Sepharose Binding Chromatographic Assay

The interaction of the antibodies with heparin was measured using a chromatographic method as described earlier [14].

### 2.7. FcRn Interaction Analyses by Surface Plasmon Resonance (SPR) and ELISA

The binding interaction of the BsAbs with recombinant cynomolgus monkey FcRn was monitored by SPR detection using a BIAcore 3000 instrument (GE Healthcare, Chicago, IL, USA) as described previously [18,19]. The binding constants (K_D_ values) at pH 6.0 were determined from the responses at equilibrium (R_eq_ values) through global fits of the average of two data sets collected on separate days using Biacore T200 Evaluation, version 1.0. Data collected at pH 7.4 were not fit since there was no observable signal.

The evaluation of the pH-dependent dissociation of BsAbs from FcRn was conducted using an ELISA as previously reported [18,19]. Briefly, biotinylated cynomolgus monkey FcRn was produced by reacting each purified soluble protein with EZ-Link^®^ Sulfo-NHS-Biotin (Pierce Chemical Co., Dallas, TX, USA) using the conditions supplied by the vendor, and the FcRn:biotin ratio was measured as 1.0 and 1.0, respectively, using the EZ™ biotin quantitation kit (Pierce). The pH-dependent ELISA for the BsAbs was performed as described in earlier studies at pH 6.0 and pH 7.4 [18,19]. Optical density (OD) data at pH 6.0 and pH 7.4 were analyzed and expressed as the total BsAb that remained bound to FcRn at pH 7.4 [(OD_pH7.4_/OD_pH6.0_) × 100%)]. The mean and standard deviation of three independent experiments were determined.

### 2.8. Cynomolgus Monkey Pharmacokinetic Study, Bioanalytical Assays and Pharmacokinetic Data Analysis

A cynomolgus monkey pharmacokinetic study for the BsAbs was conducted in accordance with the Standard Operating Procedures (SOPs) and protocols as approved by Eli Lilly and Company and in compliance with the requirements of Covance Laboratories (now Labcorp, Madison, WI, USA).

The cynomolgus monkey pharmacokinetic study was performed with male cynomolgus monkeys (2.3–3.2 kg). Three monkeys were assigned to each study group and all animals received a single intravenous (IV) bolus dose of either BsAb-1 or BsAb-2 dissolved in PBS (pH 7.4) at 5.0 mg/kg. Each animal had blood samples collected via a femoral vein. Blood samples were collected at predose, 1, 6, 12, 24, 48, 72, 96, 168, 240, and 336 h after administration of the dose. The blood samples were collected into tubes containing sodium citrate anticoagulant maintained in chilled cyroracks and centrifuged to obtain plasma. 

Concentrations of BsAb-1 or BsAb-2 in cynomolgus monkey plasma were determined using anti-human IgG ELISAs for each of the molecules as described [11,14]. Further, to determine the concentrations of BsAb-1 and BsAb-2 measured by ELISA represented the intact molecules, biotinylated goat anti-human IgG (Jackson ImmunoResearch Laboratories, Inc., West Grove, PA, USA) were used to capture the molecules at select timepoints (including 1, 72, 168, and 336 h after administration of BsAb-1 and BsAb-2) and immunoprecipitated using streptavidin coated magnetic beads. Following immunoprecipitation, samples were digested with trypsin and LC/MS was performed on digested samples. BsAb-1 and BsAb-2 quantitation was based on specific heavy chain and scFv peptides to cross-verify ELISA concentrations and determine the intactness of each molecule. There were no differences in the concentrations of BsAb-1 or BsAb-2 measured by LC/MS compared with ELISA indicating the molecules were intact.

Plasma concentration-time data following IV administration was described using a model-independent method according to the statistical moment theory using the either WinNonlin® Professional 6.3 or Phoenix^®^ WinNonlin^®^ software package (Pharsight, A Certara™ Company, St. Louis, MO, USA). The parameters calculated included the maximum serum concentration (C_max_), area under the curve (AUC_0-∞_), clearance (CL), and elimination half-life (t_1/2_). Samples with positive anti-drug antibody titers were excluded from PK parameter calculations as indicated in Figure 1.

### 2.9. Cynomolgus Monkey Radiolabel Biodistribution Study

The cynomolgus monkey distribution study was conducted in accordance with SOPs and the protocol as approved by Eli Lilly and Company and in compliance with the requirements contained in the MPI Research ((now Charles River, Mattawan, MI, USA) Radioactive Materials License Number 21-11315-02, and all applicable regulations issued by the Nuclear Regulatory Commission (NRC)) as detailed in previous studies [14]. BsAb-1 and BsAb-2 were labeled with ^125^I or conjugated to diethylene triamine pentaacetic acid (DTPA) and radiolabeled with ^111^In at MPI Research, Inc. Equal amounts of ^111^In-DTPA and ^125^I radiolabeled molecules were combined to prepare the dosing formulation containing both conjugates that was administered once using IV injection. Doses were labeled to a target dose level of 5 mg/kg (~1 mCi/kg split equally between the two isotopes).

Following dosing, blood samples were collected at 0.083, 1, 6, 24, 48, 96, and 168 h postdose (cohorts of three animals per group) and were analyzed using a gamma counter to determine the radioactivity. The %ID/g values were corrected for radioactive decay over time. Liver biopsies were collected from all animals (cohorts of three animals per group) at 1 h post administration for immunofluorescence analyses (described below). The adrenal gland, bladder (urinary), bone (femur), bone marrow (femur), brain, muscle (gastrocnemius, both quadriceps, and scapular region), heart, kidney, large intestine/cecum with contents, liver, lung, lymph nodes (mesenteric), pancreas, skin (ventral and upper and lower dorsal), small intestine with contents, spleen, stomach with contents, testes, thymus, thyroid, and fat pad were collected from one animal per timepoint per group at 48, 96, and 168 h postdose. All tissues were weighed and analyzed for total radioactivity. Urine was collected from one animal per timepoint per group from 0 to 48, 0 to 96 and 0 to 168 h postdose. A dual-isotope gamma counter setting was used for all sample measurements. Similar to the blood samples, the %ID/g values for the tissues and urine were corrected for radioactive decay over time.

### 2.10. Detection of BsAbs in Cynomolgus Monkey Liver

Liver biopsy samples from the left lobe of each animal were collected from the aforementioned biodistribution study for immunofluorescence analyses to determine the cellular disposition of BsAb-1 and BsAb-2 at 1 h post administration using previously reported methods [14]. Stored formalin fixed and paraffin embedded (FFPE) liver tissue from previously reported experiments of an ECD-based BsAb were also included as a positive control for sample processing and the detection of a molecule in tissue [14]. Briefly, liver tissues were processed to FFPE and sectioned as described in earlier studies [14]. FFPE sections were deparaffinized and rehydrated prior to immunofluorescent staining to detect BsAb-1 or BsAb-2, as well as endothelial cells (CD31 marker) [22,23]. Antigen retrieval was performed using the epitope retrieval solution, Diva Decloaker (BioCare Medical, Concord, CA, USA) for 30 s at 125 °C under pressure as reported in earlier studies [14]. Liver sections were incubated with a polyclonal anti-human IgG (Bethyl Laboratories, Montgomery, TX, USA, A80-319A) at 10 µg/mL to detect antibodies and a monoclonal anti-human CD31 (CD31/PECAM1 R&D Systems AF806) at 10 µg/mL. Additionally, species-specific control IgG obtained from Jackson ImmunoResearch, (West Grove, PA, USA) or R&D Systems, (Minneapolis, MN, USA), respectively, were used as a control to determine the specificity of BsAb detection. Following incubation with the antibodies, slides were rinsed and followed by detection with donkey anti-species Alexa dye conjugated reagents each at 10 µg/mL (Life Technologies, (Grand Island, NY, USA) or Jackson ImmunoResearch (West Grove, PA, USA). The fluorochromes used were Alexa-488 and Alexa-555.

Images of the stained slides were collected on a 3-D HISTECH (3DHISTECH Ltd., Budapest, Hungary) scanner having Plan-Aplchromat 40× objective lenses with brightness and contrast parameters consistently applied to all images as described previously [14].

## 3. Results

### 3.1. Design Rationale for the BsAbs

The BsAb-1 and BsAb-2 molecules were constructed with a scFv domain fused to the C-terminal end of the HC of a mAb via a peptide linker. A schematic of the constructs is shown in Figure 1A. The BsAbs were designed to bind to the same two soluble ligands both of which have minimal peripheral concentrations in normal cynomolgus monkeys. The BsAb molecules consisted of a target binding orientation switch, in which the Fab region (of mAb-1) of BsAb-1 binds to the same ligand as the scFv (scFv-1) component of BsAb-2. Relatedly, the Fab region (of mAb-2) of BsAb-2 binds to the same ligand as scFv-2 in BsAb-1. The orientation switch did not influence the binding to either ligand thus, both BsAbs bind to each soluble ligand with no statistically significant changes in affinity (data not shown). Each BsAb is composed of a human IgG_4_ with the same CH_1_, CH_2_, and CH_3_ regions; thus, the variable regions are the only difference between the mAbs in BsAb-1 and BsAb-2. Each of these molecules also included mutations in the Fc region, S228P/L234A/L235A (European Union (EU) numbering system), to eliminate in vivo arm exchange with endogenous IgGs and Fcγ receptor interactions [24,25]. Fusion of the scFv elements to the C-terminus of the intended IgG_4_ HC was facilitated by a flexible glycine-serine linker to generate each BsAb.

### 3.2. Physiochemical Characterization of the BsAbs

Biochemical and biophysical properties have been shown to have an influence on mAb clearance in vivo [13,26,27,28,29,30]. For the BsAbs, we evaluated these factors, which included relative solubility, isoelectric point (pI), thermal stability (T_m_), as well as the propensity for electrostatic (i.e., charge) and hydrophobicity-related interactions using heparin and hydrophobic interaction chromatography, respectively. The data from these analyses are summarized in Table 1.

Both BsAbs were readily concentrated to ~45 mg/mL in PBS buffer without any visible precipitation. Solution opalescence or turbidity provides an effective indicator of the presence of aggregates in solution or predisposition for liquid-liquid phase separation, precipitation, or aggregation [31]. To further compare the aggregation propensity of the two BsAbs, the samples were concentrated to 20 mg/mL in PBS buffer and held at 5 °C for 10 days, when turbidity was measured by a micro-turbidity assay. Both samples showed an opalescence value of ~22 NTU (nephelometric turbidity units) while negligible differences were found between them, indicating comparable aggregation propensity at this condition. The samples were further stressed by incubating at 37 °C for 7 days. Again, the samples exhibited negligible differences.

The experimental pI values for BsAb-1 and BsAb-2 were found to be 8.69 and 8.53, respectively. These pI values indicated that at physiological pH of both the BsAb molecules would be expected to have a similar weak overall positive charge. Additionally, the data indicated that the Fab/scFv conversion, or the ligand binding orientation switch between the two molecules did not grossly alter the pI (Table 1).

A previously developed heparin-based column assay was used to determine the degree of charge-based interaction for the BsAbs [12]. In this experiment, BsAb-1 and BsAb-2 were injected over a column of heparin sepharose and then eluted with a linear gradient of increasing ionic strength. Retention of the molecule and elution time was then used to determine the heparin interaction potential (HpnIP) for charge-based interactions. Due to the small positive charge present on the BsAbs, they are somewhat retained by the column, eluting at [NaCl] of 218.1 mM and 191.1 mM, respectively, but the molecules displayed similar HpnIP (24.8%HpnIP versus 22.0%HpnIP) (Table 1).

Non-specific interactions of the BsAbs driven by hydrophobic association were evaluated using a HIC-based HPLC assay in which molecules were injected onto a solid phase hydrophobic resin pre-equilibrated in high concentrations of salt. Neither BsAb was retained on the HIC column indicating that both molecules are very hydrophilic (Table 1).

Thermal stability of the BsAbs were measured by differential scanning calorimetry (DSC). Analysis of BsAb-1 and BsAb-2 indicated that the first T_m_ value of BsAb-1 is much lower than that of BsAb-2 (59.0 °C versus 67.7 °C, respectively) (Table 1 and Supplemental Figure S1). In comparing DSC thermograms of BsAb-1 vs. the matching IgG in BsAb-1, it is evident that the low Tm peak of BsAb-1 is due to the scFv portion of the molecule and that fusing the scFv domain to the C-terminal end of the HC showed no gross changes or perturbations in the thermal stability of the IgG portions (data not shown). This is consistent with our experience with other IgG-scFv fusion molecules that the IgG and scFv portions unfold independently.

### 3.3. FcRn Interaction Characterization of the BsAbs

BsAb-1 and BsAb-2 were evaluated for their cynomolgus monkey FcRn (cFcRn) binding properties using two previously reported assays [18,19]. First, the cFcRn binding affinity (i.e., K_D_ at pH 6 and 7.4) was determined using SPR. No significant difference in cFcRn binding between the two molecules was observed at pH 6. The binding affinity (K_D_) of the BsAb-1 and BsAb-2 for cFcRn at pH 6.0 were ~105 and ~115 nM, respectively (Table 2). No direct binding to cFcRn at neutral pH was detected for either of the BsAbs (Table 2). Additionally, the cFcRn binding properties of the ^125^I and ^111^In-labeled BsAbs at pH 6 and 7.4 were comparable to their unlabeled counterparts.

In the second assay, the ability of the BsAbs to dissociate from the receptor once bound was measured using an ELISA in a pH-dependent manner (Table 2) [18]. In this format, we form complexes of the each BsAb with cFcRn at acidic pH (pH 6) and measure the amount of each molecule which remains bound to cFcRn once the complex is exposed to a neutral pH (pH 7.4) as a surrogate of cFcRn intracellular binding and extracellular release; thus, the higher the percent of molecule bound to cFcRn the less efficient the release is from the receptor at neutral pH. BsAb-1 and BsAb-2 show ~54% and ~5%, respectively, and remain bound to cynomolgus monkey FcRn once the complex is exposed to neutral pH (Table 2). Radiolabeling the BsAbs did not influence the findings. Intrigued by this finding, we evaluated mAb-1 which is the mAb component of BsAb-1 and found mAb-1 showing ~3% remains bound to cynomolgus monkey FcRn under the same conditions. Evaluation of the human FcRn pH-dependent release profile for each BsAb showed similar findings to cFcRn.

### 3.4. Pharmacokinetics of the BsAbs in Cynomolgus Monkeys

The in vivo pharmacokinetic properties of BsAb-1 and BsAb-2 were examined in cynomolgus monkeys following a single IV dose of 5 mg/kg each as it was well-established that there were insignificant peripheral levels of antigen for the Fab and scFv components of BsAb to bind in normal cynomolgus monkeys. As a result, the influence of target binding on the clearance is negligible and cynomolgus monkeys served as an acceptable in vivo model to evaluation the inherent pharmacokinetics of the BsAbs.

Following administration to cynomolgus monkeys, BsAb-1 and BsAb-2 showed a clearance of ~2.0 (±0.34) mL/h/kg and ~0.23 (±0.01) mL/h/kg, respectively, and a half-life of ~40 h and ~248 h, respectively (Figure 1B and Table 3). BsAb-1 was characterized by ~10-fold faster elimination relative to BsAb-2 (Table 3). Anti-drug antibodies (ADA) were observed for both BsAb-1 and BsAb-2 later in the concentration versus time profile (>240 h post dose) (ADA titer data not shown). Since the ADA was evident at >240 h after administration, there was enough concentration versus time data to assess the clearance of the molecules and thus, the ADA did not influence the interpretation of the kinetic data. Given that the ligand binding properties for the two BsAbs are the same, the kinetic observations suggest that non-ligand mediated clearance factor(s) specifically related to the BsAb orientation are likely responsible for the in vivo pharmacokinetics.

### 3.5. Biodistribution of the Radiolabeled ^125^I- and ^111^In-DTPA- BsAb-1 and BsAb-2 in Cynomolgus Monkey Liver

**Figure 2:**
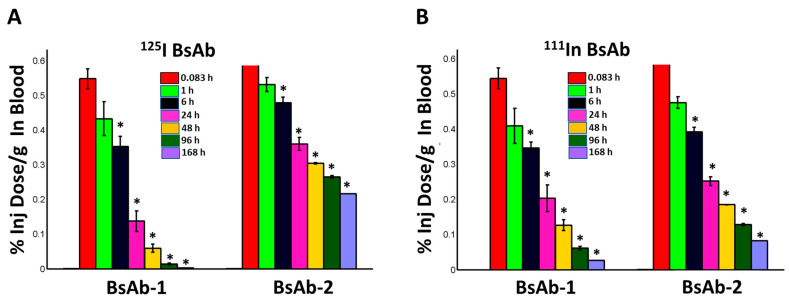
Mean concentrations (%ID/g) of (**A**) ^125^I-labeled BsAb-1 and BsAb-2 and (**B**) ^111^In- labeled BsAb-1 or BsAb-2 in male cynomolgus monkeys following a single IV administration of ~5 mg/kg (~0.015 mCi total ^125^I- and ^111^In- labeled BsAb-1 or BsAb-2 mixed in equal amounts) in blood. Blood data are the mean (+/− standard deviation or SD) for *n* = 3 timepoint for each molecule at 0083, 1, 6, 24 and 48 hours post administration for each isotope; *n* = 2 timepoint (+/− SD) for each molecule at 96 hours post administration for each isotope and *n* = 1/timepoint for each molecule at 168 hours post ad-ministration for each isotope. The SD for the *n* = 2 timepoint is displayed for illustrative purposes only. Statistical comparisons of the concentration versus time data were conducted between the two molecules labeled with the same isotope and between the same timepoint after administration. The * symbol indicates statistically significant (*p* value < 0.05) differences between BsAb-1 and BsAb-2 for the same isotope at the same timepoint post administration.

**Figure 3:**
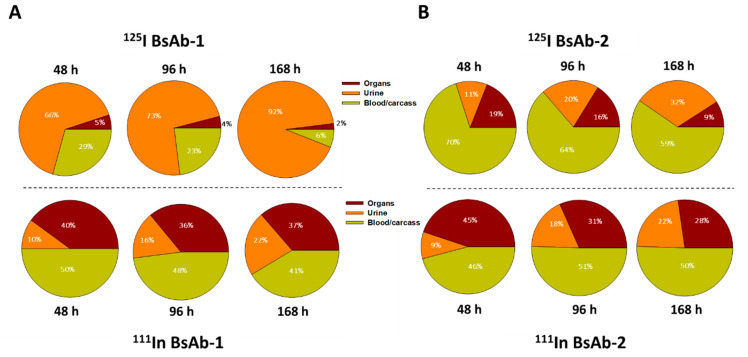
Percent of radioactive recovery data for (**A**) BsAb-1 and (**B**) BsAb-2 in male cynomolgus monkeys following a single IV administration of ~5 mg/kg (~0.015 mCi ^125^I- and ^111^In- labeled BsAb-1 or BsAb-2 mixed in equal amounts) in the organs, urine, blood and carcass. Organs include liver, spleen, kidney, lung, skin, muscle and other tissue collected as per outlined in Materials and Methods section. Data are for *n* =1/timepoint for each isotope.

The biodistribution, including the tissue elimination and tissue accumulation kinetics of BsAb-1 and BsAb-2, were studied in cynomolgus monkeys to gain additional mechanistic insight into the differential peripheral clearance observations. Following a single IV injection of ^125^I-labelled and ^111^In-labelled DTPA conjugated versions of the two BsAb molecules, blood, tissue (organ) and carcass concentrations were measured for each molecule over the course of 168 h post administration (Figure 2 and Figure 3). In addition, urine samples were also collected to calculate the total amount of radioactive recovery to monitor the elimination kinetics (Figure 3). The reason for using two different versions of each molecule was that the ^125^I-labelled and ^111^In-labelled DTPA conjugated BsAbs have differential retention in tissues. In the case of the radiohalogen (^125^I), catabolic products are cleared from cells; hence, this labeling approach provides the rate of clearance of each BsAb from tissue and the amount of catabolic products recovered in urine. In contrast, the catabolism of the radiometal-chelate (^111^In-labelled DTPA) leads to the catabolite being trapped in cells because of the polar nature of chelate; given this, the labeling approach provides the extent of accumulation of the BsAbs in tissues.

Radiometrically derived blood kinetics (with both labels) showed the clearance of BsAb-1 > BsAb-2 (Figure 2) consistent with the exposure profiles of the unlabeled counterpart of each construct suggesting labeling the molecules did not impact the disposition. Quantitative analyses of the rate and the extent of accumulation of the ^111^In-labeled BsAbs in the major highly vascularized tissues (liver, spleen, kidney, lung, skin, muscle, and other tissue collected as per outlined in Materials and Methods section) showed no meaningful differences in how fast or how much BsAb-1 and BsAb-2 accumulated in these tissues (refer to combined organ data from ^111^In-labeled BsAb-1 and BsAb-2 data in Figure 2). Liver had the highest percentage of the injected dose of each molecule relative to the other tissues (data not shown); however, consistent with the combined ^111^In-labeled organ data, no difference in the tissue accumulation kinetics (%ID/g from ^111^In-labeled analyses) or amount (%ID/g from ^111^In-labeled analyses) between BsAb-1 and BsAb-2 was observed (Figure 2). In contrast, quantitative analyses of the rate and extent of elimination of the ^125^I-labeled BsAbs in the major highly vascularized tissues (liver, spleen, kidney, lung, skin, muscle, and other tissue collected as per outlined in Materials and Methods section) showed striking differences in how fast BsAb-1 and BsAb-2 were cleared from the tissues and catabolic products were measurable in urine (refer to data from ^125^In-labeled BsAb-1 and BsAb-2 data in Figure 2). BsAb-1 showed ~3- to 6 times greater concentrations of ^125^I in urine than BsAb-2 over time (Figure 3).

Comparison of the biodistribution data from the two labels for BsAb-2 over time shows that the findings are reasonably similar for ^125^I-BsAb-2 and ^111^In-DTPA-BsAb-2 (Figure 2 and Figure 3). The trend for higher ^125^I-BsAb-2 concentrations relative to ^111^In-DTPA-BsAb-2 in the blood is most likely related to the retention of the radiometal within tissues. However, the data for both of the labels indicates BsAb-2 is largely in blood as there was ~2- to 3-fold increase (or retention) of the molecule in the organs with the ^111^In-DTPA conjugation compared with ^125^I-label over time (Figure 3). Additionally, a larger portion of the ^125^I-label percent recovery data was in the organs and blood relative to urine for BsAb-2 over time (Figure 3). The low urinary elimination and larger proportion of molecule in the blood over time in the case of both labels for BsAb-2 supports the contention that a large proportion of BsAb-2 taken into tissues is recycled back into blood. In contrast to the BsAb-2 results, for BsAb-1 which showed more rapid peripheral clearance (Figure 1B and Figure 3), there are striking differences in the findings between ^125^I-BsAb-1 and ^111^In-DTPA-BsAb-1 (Figure 3). The data for the two labels indicates BsAb-1 is largely retained in the organs as there is ~7- to 18-fold increase (or retention) of the molecule in the organs with the ^111^In-DTPA conjugation compared with ^125^I-label over time (Figure 3). Additionally, a larger portion (65% to 92%) of the ^125^I-BsAb-1 percent recovery data was in the urine compared with the organs and blood over the time course (Figure 3). Taken together, these data indicate that the two BsAbs are taken up into tissue to a comparable extent and rate but, there is a more rapid catabolism of BsAb-1 than BsAb-2 in tissues and elimination of catabolite products in the urine.

### 3.6. Liver Tissue Distribution of the BsAbs in Cynomolgus Monkey Using Immunofluorescence

**Figure 4:**
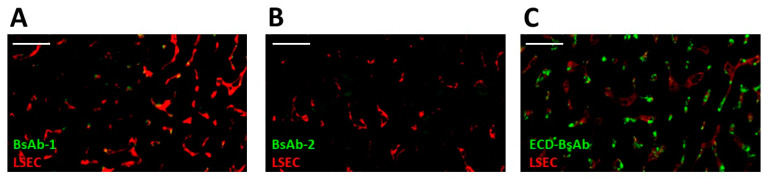
Immunofluorescence of BsAb-1, BsAb-2 and an ECD-based BsAb in cynomolgus monkey liver following a single IV administration of each molecule. In each panel green and red represent detection of the BsAb (using an anti-human (IgG) and LSECs (via detection of the endothelial cell marker CD31 PECAM1), respectively. Immunofluorescence detection of (**A**) BsAb-1 (**B**) BsAb-2 and (**C**) ECD-based BsAb (used as a positive control from a previous study) [13]. Data are from representative liver sections from a single cynomolgus monkey. The scale bars represent 50 um.

The biodistribution study revealed that BsAb-1 and BsAb-2 were distributed to the liver (Figure 4). Previous reports from our laboratories showed BsAbs that have rapid peripheral clearance can show multifocal labeling of the sinusoidal lining around the hepatocytes consistent with endothelial cell association [11,14]. In an effort to delineate if the clearance difference between BsAb-1 and BsAb-2 were mechanistically a consequence of liver sinusoidal endothelial cells (LSEC), immunofluorescence detection of the BsAbs was evaluated at 6 h following a single 5 mg/kg IV administration of each molecule on liver biopsies collected from the aforementioned cynomolgus monkey radiolabel biodistribution study. This timepoint was chosen based on the cynomolgus monkey pharmacokinetic profiles displaying a reasonable divergence in the peripheral exposure of the BsAbs (Figure 1B). Additional identification of LSECs was pursued by immunofluorescence detection of CD31, which are commonly used to identify endothelial cells in liver. Stored liver tissue sections from a previously reported ECD-based BsAb which displayed liver deposition were included in the immunofluorescence staining as a positive control to provide a benchmark of LSEC association for interpretation of BsAb-1 and BsAb-2 data [14]. At 6 h post administration, neither BsAb-1 or BsAb-2 were detected in a distinguishable manner in the liver, but the positive control ECD-based BsAb was clearly detected (Figure 4). The data suggest that liver sinusoidal endothelial cells are not linked to the rapid clearance of BsAb-1 relative to BsAb-2.

## 4. Discussion

During our PK characterization of two related scFv-based BsAb constructs, we observed an unusually rapid ~10-fold faster clearance of BsAb-1 relative to BsAb-2. Both BsAb-1 and BsAb-2 constructs were fabricated with scFv domains fused to the HC C-terminal of IgG_4_ mAbs. The two BsAbs displayed comparable binding affinity to the same soluble ligands that had no/minimal peripheral concentrations in normal animals; moreover, the BsAbs had no specific interaction with cell surface receptors thus, eliminating both circulating ligand-mediated and cell surface target-mediated drug disposition as potential mechanisms for the observed clearance. In addition, since both BsAbs examined use an IgG_4_ parental Fc engineered to eliminate interactions with Fcγ receptors, direct binding with blood cells is not expected to be a viable clearance mechanism either. Given the disparity in clearance was not related to target binding or Fcγ receptor interactions, the focus of the present effort became delineating the non-target-related physiological mechanism(s) affecting the BsAb in vivo behavior.

Non-target-related physiological factors influencing the disposition and PK of BsAbs are poorly understood. In previous reports we demonstrated that the association with liver sinusoidal endothelial cells (LSECs) led to the accumulation of BsAbs in liver and was responsible for the unusually rapid clearance of several BsAbs including some IgG-scFv constructs [14]. Given these findings, LSEC clearance became an initial plausible mechanism to explore for BsAb-1 and BsAb-2 that can readily explain the atypical elements of the BsAb clearance. Interestingly, the IHC analyses of liver from cynomolgus monkey PK studies conducted with the BsAbs showed that LSECs were not the root cause of the rapid clearance of BsAb-1 relative to BsAb-2 (Figure 4). These negative IHC data were also consistent with radiolabel biodistribution studies in cynomolgus monkeys, which showed that BsAb-1 and BsAb-2 had a similar rate and extent of distribution to tissues (Figure 2). The similar rate and extent of accumulation in the tissues observed for BsAb-1 and BsAb-2 indicate comparable tissue disposition for the molecules and by extension, that the PK differences were not due to clearance via a specific organ. The key difference noted in the biodistribution studies of BsAb-1 and BsAb-2 was the rate of clearance of the molecules from the major organs of clearance. The increased clearance of BsAb-1 relative to BsAb-2 from all the major tissues of elimination along with the greater amount of catabolite recovery of BsAb-1 in urine, strongly suggests that following uptake into tissues BsAb-1 was not efficiently recycled back to the blood circulation and instead degraded. Given these data, we speculated other plausible mechanisms, such as physiochemical properties leading to differential molecular stability, non-specific binding profiles and/or FcRn interactions, may be causative and account for the rapid tissue elimination of BsAb-1.

Physiochemical properties including positive charge, poor thermal stability, and hydrophobic-based interaction potential that can facilitate non-specific interactions have been linked to the PK developability of mAbs [8,9,10]. While there are a paucity of such studies for BsAbs, in previous reports we found that increased global structural stabilization and reduced hydrophobicity were connected with in vivo kinetics for BsAbs with an ECD format [11]. In the case of BsAb-1 and BsAb-2 in this report, the molecules have largely comparable physiochemical profiles, with BsAb-1 exhibiting lower first melting temperature than BsAb-2 (Table 1). Nevertheless, the lower first melting temperature in BsAb-1 is much higher than the physiological temperature (37 °C) and a T_m_ in this range by itself is not known to cause aberrant clearance [11,14]. Furthermore, the lower T_m_ does not lead to increased aggregation at a relatively high concentration of 20–45 mg/mL. Taken together, the physiochemical differences are relatively small, and we speculate that these factors alone are not major contributors to the differential clearance of BsAb-1 and BsAb-2.

Another plausible mechanism that we and others have suggested which can negatively affect the PK of mAbs (and by extension BsAbs) is the FcRn interaction profile [17,18,19]. The FcRn interaction profile includes the direct binding interactions of molecules to FcRn at both an acidic (pH 6) and neutral pH (pH 7.4), as well as characterization of the rate of the dissociation of the IgG:FcRn complex as the pH increases. The later parameter contextualizes interactions with FcRn which would impede mAb recycling and release within the endosomal compartment and into the peripheral circulation [18,27,32]. In vitro analyses of BsAb-1 and BsAb-2 using previously published approaches showed that BsAb-1 and BsAb-2 bound to FcRn similarly at pH 6 and showed no binding to FcRn at pH 7.4 (Table 2). These data indicated that direct FcRn binding was unlikely to be related to the differential clearance observations between BsAb-1 and BsAb-2. However, additional characterization of the FcRn release profile for each BsAb showed striking differences. In the FcRn release assessment, complexes of each of the BsAb with FcRn at acidic pH (pH 6) were formed and the amount of BsAb which remained bound to FcRn once the complex was exposed to neutral pH (pH 7.4) was measured as a surrogate of FcRn intracellular binding and extracellular release activities. There is an ~8 times larger amount of BsAb-1 that remained bound to FcRn once the complex was exposed to neutral pH compared with BsAb-2, indicating that BsAb-1 is less efficiently released from FcRn upon the pH change (Table 2). Given the ~20,000- to 45,000-fold lower concentration of the samples in the FcRn dissociation experiment (0.001 mg/mL) than that used in the solubility studies (20–45 mg/mL) where no significant difference between the two BsAbs was observed, it is highly unlikely that BsAb-1 aggregation contributed to the inefficient release of BsAb-1 from FcRn. Previous studies with mAbs have also implicated altered pH-dependent release from FcRn as a causative mechanism for the rapid clearance and short half-life in animals [18,19]. Studies with mAbs have shown that altered release from FcRn can affect both the distribution phase (α phase) and the elimination phase (β phase) of the kinetic time course [18,19,33]. The PK profile of BsAb-1 showed a rapid distribution and short half-life consistent with the hallmarks of an altered FcRn release at neutral pH, further supporting that poor release from FcRn is likely the perpetrator mechanism for the poor PK behavior of BsAb-1. Thus, taken together with these noted observations for mAbs, the poor FcRn release profile contributes to the more rapid in vivo clearance of BsAb-1 and is very likely the major culprit mechanism for the PK difference observed relative to BsAb-2. Interestingly, analyses of the FcRn release profile of the parental mAb (mAb-1) used to construct BsAb-1 did not show any evidence of poor dissociation from the receptor at neutral pH (refer to Results section). This suggests that the fusion of scFv-2 to mAb-1 altered the FcRn interactions of BsAb-1, which was not the case when scFv-1 was fused to mAb-2 to construct BsAb-2.

**Figure 5:**
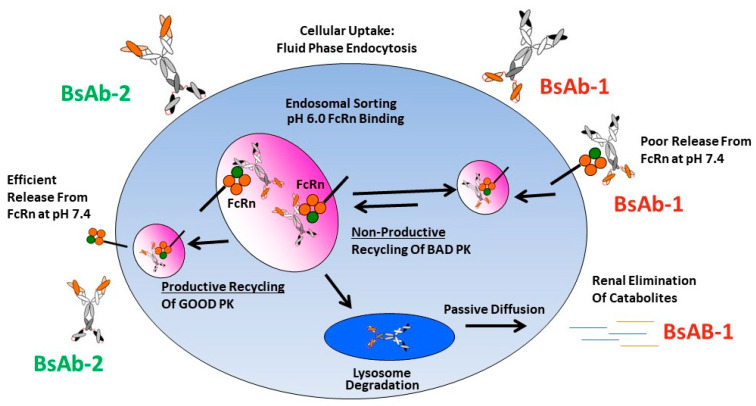
Mechanistic speculation of the intracellular trafficking pathways for BsAb-1 and BsAb-2 which lead to differential peripheral clearance.

While dysfunctional FcRn interactions have been noted to negatively impact mAb PK, previous studies of other BsAbs showed no connectivity to FcRn as causative in rapid clearance observations [11,14,16,34,35,36]. To the best of our knowledge, the data presented herein is the first report connecting an altered FcRn release to BsAb PK. We speculate that there are likely differences in the intracellular trafficking of BsAb-1 and BsAb-2 linked mechanistically with FcRn-mediated recycling that connect to the in vivo catabolism, elimination and PK observations for the two molecules (Figure 5). We postulate that at the cellular level the molecules are largely comparably internalized via fluid phase endocytosis into endosomes which facilitates binding FcRn within the acidic environment of this compartment. The relative similarity in the BsAb-1 and BsAb-2 physiochemical and direct FcRn binding properties (at acidic and neutral pH) are consistent with this proposed non-specific intracellular internalization and endosomal FcRn binding mechanisms. Next, in the case of BsAb-2, the in vitro and in vivo data indicate the molecule likely undergoes ‘productive recycling’ which is connected to antibody-based biologics with acceptable PK developability. In this situation, intracellular BsAb-2 is mostly salvaged from lysosomal degradation by FcRn-mediated recycling. Indeed, the efficient release from FcRn at neutral pH in vitro supports the molecule’s PK profile. Additionally, the slowed blood clearance of BsAb-2 supports the molecule being productively recycled back into the blood circulation once the receptor:BsAb complex is exposed to the neutral pH outside cells. In contrast, for BsAb-1 which displayed poor PK, we hypothesize that the molecule undergoes ‘non-productive recycling’ whereby when the FcRn:BsAb complex is exposed to neutral pH there is inefficient release of BsAb-1 from FcRn. The inefficient release may shift the trafficking equilibrium such that BsAb-1 is not released from cells and eventually degraded. The ~54% of BsAb-1 that remained bound to FcRn at a neutral pH in vitro is consistent with this speculation (Table 2). Along those lines, the greater rate and extent of BsAb-1 catabolites found in urine (relative to BsAb-2) is also supportive of the proposed mechanism (Figure 3). Additional interrogation of our BsAb constructs using other approaches including cell-based trafficking and imaging studies may provide further insight in future studies.

While a more complete understanding of the complex matter of PK developability factors for BsAbs will undoubtedly require additional interrogation beyond the limited number of constructs presented herein, this report contributes to the growing body of literature of how multiple physiochemical and biochemical factors influence the peripheral clearance and disposition of BsAbs. Even in the case of mAbs, the precise relationship between their physiochemical and biochemical properties and an a priori prediction of in vivo PK and disposition remains elusive. Many labs (including ours) have demonstrated evidence to connect nonspecific-binding propensity, hydrophobicity, and charged regions in molecules to rapid clearance in both preclinical species and humans [11,12,13,37]. Furthermore, in a comprehensive study of 137 antibodies in a dozen biophysical property assays, Jain and coworkers reported there appeared no single unfavorable assay that definitively predicted the failure to advance molecules to clinical trials and supported a more holistic approach of multiple biophysical properties on a molecule specific basis [10]. Interestingly, when we benchmarked the biophysical property assessments of the BsAbs herein to those reported by Jain et al., our BsAbs were not considered as undesirable from a PK developability perspective, although there were no measures of evaluation of the pH-dependent dissociation from the FcRn reported [10]. Given the altered FcRn release appeared to most readily connect to the PK differential between the two BsAbs in this report, it is possible that the parameterization of ‘favorable’ and ‘unfavorable’ physiochemical guardrails for BsAb disposition may be different from mAbs and require this factor as an additional consideration.

In summary, the findings in this report are an important demonstration that BsAb PK can be impacted by a variety of factors. There are multiple PK developability considerations including the nature of the BsAb targets (target/turnover/tissue distribution), the physiochemical and biochemical properties of the BsAbs, and the BsAb structural configuration that can influence disposition and elimination differentially. As more BsAb structures advance into clinical studies, careful case-by-case physiochemical and biochemical assessments along with the connectivity of these to the in vivo PK/disposition are critical for their continued advancement as therapeutic modalities. Careful delineation of preponderance of these factors on a molecule-to-molecule basis can ultimately lead to the selection and design of BsAbs with increased therapeutic value for patients.

## Figures and Tables

**Table 1:** Physiochemical Properties of the BsAbs.

Molecule	Heparin Chromatography		HIC Chromatography	Measured pI	Calculated pI		DSC
	Elution NaCl (mM)	%HpnIP		BsAb	Fab	scFv	First T_m_ (°C)
BsAb-1	218.1	24.8	No retention	8.69	8.37	7.79	59.0
BsAb-2	191.1	22.0	No retention	8.53	7.94	8.28	67.7

**Table 2:** FcRn Interaction Properties of the BsAbs.

Molecule	FcRn K_D_ (nM) at pH 6 *	FcRn K_D_ (nM) at pH 7.4 *	Percent Bound at pH 7.4 ^^^
BsAb-1	105 ± 15	No binding	53.8 ± 9.8
BsAb-2	115 ± 25	No binding	4.6 ± 0.2

* FcRn K_D_ represents cynomolgus monkey FcRn binding affinity at pH 6. No direct binding to FcRn was observed at pH 7.4 at a concentration of 5 mM for both BsAb-1 and BsAb-2. ^^^ Percentage of the total molecule FcRn:BsAb complexes preformed at pH 6 that remained FcRn-bound at pH 7.4 as determined by ELISA.

**Table 3:** Cynomolgus Monkey Pharmacokinetic Parameters of the BsAbs following a single IV administration of 5 mg/kg.

Molecule	C_max_ (μg/mL)	T_max _ (h)	AUC_0-inf_ (h·μg/mL)	CL or (mL/h/kg)	V_ss_ (mL)	T_1/2_ (h)
BsAb-1	100 ± 15	1 ± 0	2566 ± 439	1.99 ± 0.34	77 ± 11	40 ± 3
BsAb-2	168 ± 47	1 ± 0	21628 ± 552	0.23 ± 0.01	79 ± 2	248 ± 2

C_max_: maximal observed serum concentration; T_max_: time of maximal observed serum concentration; AUC_0-inf_: area under the serum concentration curve from time zero extrapolated to infinite time; CL: clearance; T_1/2_: elimination half-life; V_ss_: volume of distribution at steady state. Data are the mean (±standard deviation) from *n* = 3/animals.

## Data Availability

Data may be made available upon request.

## Supplementary Materials

The following are available online at https://www.mdpi.com/article/10.3390/antib11010002/s1, Figure S1: DSC thermograms of (A) BsAb-1 and (B) BsAb-2 in PBS, pH~7.2. All domains in BsAb-2 unfold at similar temperatures so the individual domain transitions are unresolved. The total ΔH for BsAb1 and BsAb2 are similar.
