# Supplementary material for: Pharmacokinetic Developability and Disposition Profiles of Bispecific Antibodies: A Case Study with Two Molecules
Source: Antibodies (Basel). 2021 Dec 28;11(1):2. doi: 10.3390/antib11010002 (PMC8788489; doi:10.3390/antib11010002)

Figure S1: DSC thermograms of (A) BsAb-1 and (B) BsAb-2 in PBS, pH 7.2. All domains in BsAb-2 unfold at similar temperatures so the individual domain transitions are unresolved. The total ΔH for BsAb1 and BsAb2 are similar.

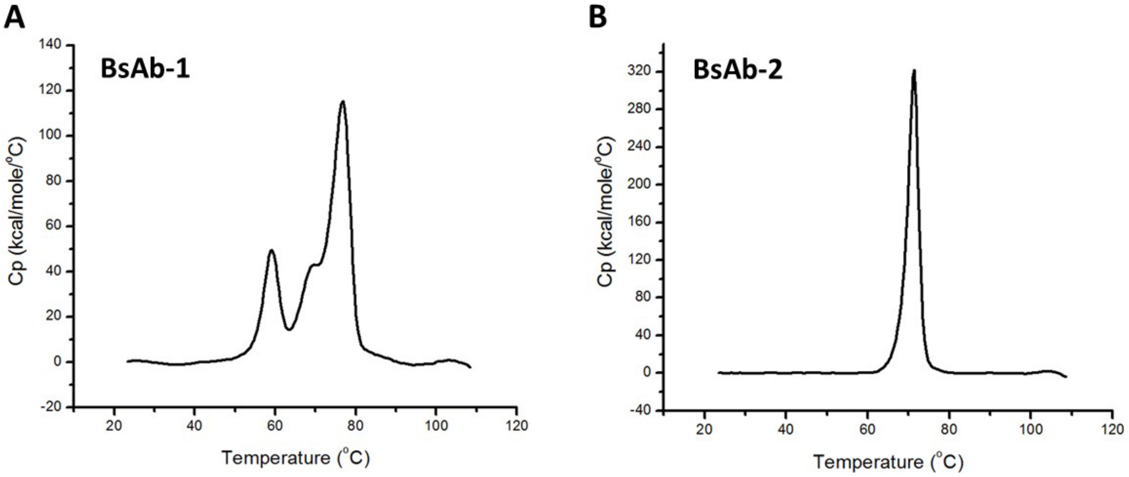

Supplement: Supplementary file 1 [file antibodies-11-00002-s001.zip › antibodies-1395795-supplementary.pdf]
